# Inhibition of macropinocytosis blocks antigen presentation of type II collagen *in vitro *and *in vivo *in HLA-DR1 transgenic mice

**DOI:** 10.1186/ar1964

**Published:** 2006-05-16

**Authors:** Alexei von Delwig, Catharien MU Hilkens, Daniel M Altmann, Rikard Holmdahl, John D Isaacs, Clifford V Harding, Helen Robertson, Norman McKie, John H Robinson

**Affiliations:** 1 Musculoskeletal Research Group, Clinical Medical Sciences, University of Newcastle upon Tyne, Framlington Place, Newcastle upon Tyne, UK; 2 Human Disease Immunogenetics Group, Department of Infectious Diseases, Imperial College School of Medicine, Hammersmith Hospital, London, UK; 3 Department of Cell and Molecular Biology, Lund University, Lund, Sweden; 4 Department of Pathology, Case Western Reserve University, Cleveland, OH, USA; 5 BioImaging Facility, Clinical Laboratory Sciences, University of Newcastle upon Tyne, Framlington Place, Newcastle upon Tyne, UK

## Abstract

Professional antigen-presenting cells, such as dendritic cells, macrophages and B cells have been implicated in the pathogenesis of rheumatoid arthritis, constituting a possible target for antigen-specific immunotherapy. We addressed the possibility of blocking antigen presentation of the type II collagen (CII)-derived immunodominant arthritogenic epitope CII_259–273 _to specific CD4 T cells by inhibition of antigen uptake in HLA-DR1-transgenic mice *in vitro *and *in vivo*. Electron microscopy, confocal microscopy, subcellular fractionation and antigen presentation assays were used to establish the mechanisms of uptake, intracellular localization and antigen presentation of CII by dendritic cells and macrophages. We show that CII accumulated in membrane fractions of intermediate density corresponding to late endosomes. Treatment of dendritic cells and macrophages with cytochalasin D or amiloride prevented the intracellular appearance of CII and blocked antigen presentation of CII_259–273 _to HLA-DR1-restricted T cell hybridomas. The data suggest that CII was taken up by dendritic cells and macrophages predominantly via macropinocytosis. Administration of amiloride *in vivo *prevented activation of CII-specific polyclonal T cells in the draining popliteal lymph nodes. This study suggests that selective targeting of CII internalization in professional antigen-presenting cells prevents activation of autoimmune T cells, constituting a novel therapeutic strategy for the immunotherapy of rheumatoid arthritis.

## Introduction

Professional antigen-presenting cells (APCs), such as dendritic cells (DCs), macrophages and B lymphocytes, play a pivotal role in the pathogenesis of autoimmune diseases in animal models by presenting arthritogenic T cell epitopes to autoimmune T cells [1-3] Adoptive transfer of *ex vivo *cultured autoantigen-specific DCs has been shown to induce a variety of experimental autoimmune diseases, such as autoimmune diabetes, experimental autoimmune encephalomyelitis and erosive inflammatory arthritis [4-6]. DCs *in situ *are often surrounded by a cluster of T cells [7] and are thought to internalize autoantigens from the extracellular matrix and cartilage for intracellular processing and presentation of arthritogenic epitopes to specific CD4 T cells, as well as to induce activation of B lymphocytes in patients with rheumatoid arthritis (RA) [8]. B lymphocytes have also been shown to be critical both as antigen-presenting and antibody-secreting cells in the pathogenesis of autoimmune arthritis [3,9-11] High efficiency of antigen presentation of arthritogenic epitopes by macrophages has also been demonstrated [12,13].

Type II collagen (CII, α1(II)3), the most abundant fibrillar protein of articular cartilage [14], is considered an important autoantigen involved in the pathogenesis of collagen-induced arthritis in mice and RA in humans [15,16] In addition, collagen has been shown to deliver a direct maturation stimulus to DCs [17], possibly via ligation of Toll-like receptor 4 (TLR4) or by binding to cell surface integrins [18,19], suggesting that DCs can present collagen T cell epitopes without additional inflammatory or danger signals. A direct co-stimulatory activity of collagen has, however, not been demonstrated *in vivo *as there is no evidence that collagen by itself displays adjuvant activity [20]. No information is available on the mechanisms engaged in CII uptake into professional APCs for presentation of arthritogenic epitopes to CD4 T cells.

Several mechanisms have been described to mediate internalization of antigens into APCs, including phagocytosis, macropinocytosis, receptor-mediated endocytosis and caveolar endocytosis. Phagocytosis follows the recognition of particles ≥0.25 μm by specific receptors and an F-actin microfilament-dependent internalization into phagosomes [21]. Macropinocytosis does not require ligation of specific receptors and is accompanied by membrane ruffling and F-actin-dependent uptake into large macropinosomes of 0.15 to 5.0 μm [22]. Receptor-mediated endocytosis of smaller particles and molecules engages clathrin-coated pits and F-actin recruitment at endocytic sites [23], while clathrin-independent endocytosis is dependent on intact caveolae and lipid rafts [24]. In contrast to other internalization mechanisms, caveolar endocytosis does not deliver antigens to lysosomes and, therefore, does not appear to play a major role in antigen processing and presentation [2,25].

In this report, we show that CII was taken up preferentially via macropinocytosis into DCs and macrophages from HLA-DR1 transgenic mice for antigen presentation of both the glycosylated and non-glycosylated forms of the arthritogenic CII_259–273 _epitope to CD4 T cells. Treatment of mice with an inhibitor of macropinocytosis also prevented activation of CII_259–273_-specific T cells *in vivo*.

## Materials and methods

### Antigens

Human CII purified from normal human cartilage was purchased from MD BioSciences (Zürich, Switzerland). The glycosylated peptide (GIAGF KGEQGPKGET; K = GalHyL_264_) corresponding to epitope CII_259–273 _GalHyL_264 _was synthesized using β-D-galactopyranosyl-5-hydroxy-L-lysine, as described previously [26]. The non-glycosylated peptide pCII_259–273 _was purchased from GenScript Corp. (Piscataway, NJ, USA), and purity was confirmed by high-performance liquid chromatography.

### Animals

In all experiments described in this study we used previously reported mice transgenic for HLA-DR1 on a major histocompatibility complex (MHC) class II-deficient background (designated C57BL/6J^0-0 ^HLA-DR1), which carried full-length genomic constructs for HLA-DRA1*0101 and HLA-DRB1*0101, developed by one of us (DMA) [27]. Experiments described in this report have been performed under the terms of Animals (Scientific Procedures) Act 1986, and authorized by the Home Secretary, Home Office UK. The work has been approved by the Ethical Review Committee of the University of Newcastle upon Tyne.

### Cells

Culture media ingredients and inhibitors were purchased from Sigma Chemical Co. (Dorset, UK), unless stated otherwise. Cells were grown in culture medium (RPMI 1640 medium containing 3 mM L-glutamine, 50 μM 2-mercaptoethanol, 10% FBS and 30 μg/ml gentamycin). T cell hybridomas HCII-9.1 (specific for the non-glycosylated peptide) and HCII-9.2 (specific for the glycosylated peptide) have been described previously [27]. Macrophages were grown from femoral bone marrow cells in culture medium supplemented with 5% horse serum, 1 mM sodium pyruvate, 10 mM HEPES and 7.5% of a supernatant from the L929 cell line as a source of macrophage colony stimulating factor (M-CSF), as described [27]. Macrophages were activated with 10 U/ml recombinant IFN-γ (R&D Systems, Abingdon, UK) for 24 hours (purity approximately 95% based on CD11b expression).

Dendritic cells were grown from bone marrow progenitor cells in the culture medium supplemented with 20 ng/ml recombinant mouse granulocyte-macrophage colony stimulating factor (GM-CSF; BioSource International, Nivelles, Belgium) for 5 days with culture medium changes on days 2 and 3. On day 5, DCs were purified using CD11c-labeled magnetic MicroBeads (Miltenyi Biotec, Bisley, Surrey, UK), according to the manufacturer's instructions (purity approximately 92% based on CD11c expression). Maturation was induced by treatment of DCs with 0.2 μg/ml lipopolysaccharide (LPS; purified by phenol extraction from *Salmonella enterica*, serovar *typhimurium*, Sigma Chemical Co.) for 24 hours.

### Antigen presentation assays

Adherent macrophages at 10^5^/well in 48 flat-well plates (Corning Limited, Artington, Surrey, UK), or mature and immature DCs at 10^4^/well in flat-bottomed 96 well plates (Greiner Bio-One Ltd, Stonehouse, Gloucestershire, UK) were pulsed with a dilution series of CII or relevant synthetic peptides (range 40.0 to 0.02 μg/ml) for 5 hours in the absence or presence of inhibitors of uptake (10.0 μM cytochalasin D, 5.0 μM monodansylcadaverine, 1.0 mM amiloride, 0.2 mM 5-(N,N-dimethyl) amiloride (DMA) or 0.4 μg/ml filipin) for 5 hours at 37°C [28,29]. Time and the optimal doses of APCs, antigens and inhibitors were established in separate dose-response experiments. Cells were fixed with 1.0% paraformaldehyde for 5 minutes, washed thoroughly to remove the fixative and T cell hybridoma HCII-9.1 (specific for the non-glycosylated epitope) and HCII-9.2 (specific for the glycosylated epitope) were added (5 × 10^4^/well) and incubated for 24 hours at 37°C. Usage of synthetic peptides in all experiments controlled for the non-specific toxic effect of metabolic inhibitors and the responsiveness of T cell hybridomas. The interleukin-2 content of hybridoma supernatants was measured by bioassay as the proliferative response of the cytotoxic T cell line-2 (3 × 10^4^/well; CTLL-2, ATCC, TIB 214, American Type Culture Collection, Rockville, MD, USA). Proliferation assays were performed by incubating popliteal lymph node cells or spleen cells (2 × 10^5^/well) with a dilution series of CII and synthetic peptides for 72 hours, as previously described [30].

Cells were incubated during the last 18 hours in the presence of 14.8 kBq of [^3^H]thymidine (TRA310, specific activity 307 MBq/mg; Amersham International plc, Didcot, Oxfordshire, UK), harvested on glass fiber membranes and radioactivity was quantified using a direct Beta Counter (Matrix 9600, Packard Instrument Company, Meridan, CT, USA).

### Proliferation assays

For testing CII-specific T cell responses in draining lymph nodes, mice were immunized in the footpad with 50 μg CII emulsified 1:1 in TiterMax adjuvant in the absence or presence of amiloride (150 μg/mouse [31]) and popliteal lymph nodes were removed 7 days later. Cells (2 × 10^5^/well) were mixed with a dilution series of CII, synthetic peptides or the polyclonal T cell mitogen concanavalin A in round-bottomed 96 well plates (Corning Limited) and incubated for 4 days at 37°C in a humidified CO_2 _incubator. Cells were incubated during the last 18 hours in the presence of 14.8 kBq of [^3^H]thymidine, harvested and radioactivity was measured, as described above.

### Subcellular fractionation

Macrophages (15 to 20 × 10^6 ^cells) were pulsed with 200 μg/ml CII for 30 minutes and chased for different periods of time. Macrophages were homogenized in buffer containing 0.25 M sucrose, 10 mM HEPES, pH 7.4 in a Dounce tissue grinder (Wheaton, Millville, NJ, USA) to obtain 80% to 85% cell lysis. Subcellular fractionation of macrophages was performed by density gradient centrifugation in 27% Percoll (Amersham plc, Little Chalfont, Buckinghamshire, UK) using a Sorvall type A-1256 fixed angle rotor (36,000 × g, 60 minutes, 4°C; Kendro Laboratory Products plc, Bishop's Stortford, Hertfordshire, UK), as described previously [27]. Six fractions of 1.5 ml were collected manually numbered 1 to 6 from the top of the gradient. Percoll gradient fractions were each tested for β-hexosaminidase activity (marker for the presence of lysosomal enzymes) and alkaline phosphodiesterase I activity (marker for the presence of plasma membranes), as described [27,32] The localization of markers of endosomal compartments and CII within subcellular fractions was performed by ELISA, as previously described [33]. Briefly, 50 μl of Percoll fractions were dried in a constant flow cabinet in 96 well Microtiter^® ^Immunoassays plates (Immulon^® ^1, flat bottom, Dynex Technologies, Southampton, UK). Plates were blocked in phosphate-buffered saline containing 0.05% Tween 20, 10% FBS and unlabeled anti-mouse mAb specific for FcγIIR and FcγIIIR (1:200; clone 2.4G2, Fc Block^®^, PharMingen, Oxford, UK) for 1 hour at room temperature. Plates were washed and incubated for 1 hour with goat anti-CII polyclonal antibody, goat anti-Rab7 and Rab9 polyclonal antibody (1: 200; Santa Cruz Biotechnology, Inc., Heidelberg, Germany). Normal goat serum was used in control experiments. After washing, plates were incubated for 1 hour with rabbit anti-goat IgG peroxidase conjugate diluted 1:1000, washed and the reaction was developed with the liquid substrate system for ELISA 2,2'-azino-bis(3-ethylbenzthiazoline-6-sulfonic acid. Absorbance was measured at 405 nm.

### Flow cytometry

Bone-marrow macrophages and DCs were incubated in the absence or presence of inhibitors of uptake for five hours, and the expression of HLA-DR, CD80, CD86 and CD40 molecules was analyzed by flow cytometry, as described [28]. Briefly, cells were incubated for 30 minutes at 4°C in Hank's balanced salt solution containing 2% FBS, 0.01 M HEPES buffer with purified anti-mouse CD16/CD32 (Fc Block^®^, BD-PharMingen) followed by incubation for 30 minutes at 4°C with either of the following mAb fluorescent conjugates (BD-PharMingen, Cowley, Oxford, UK): anti-HLA-DR FITC, anti-CD40 FITC, anti-CD80 PE, anti-CD86 FITC, anti-CD11c FITC, anti-CD11b FITC or isotype control, rat IgG2a PE plus IgG2b FITC. Cells were analyzed with a FACScan^® ^flow cytometer (Becton Dickinson, Mount View, CA, USA), and 10,000 events were collected for each sample.

### Electron microscopy

Bone-marrow macrophages and DCs were pulsed with 200 μg/ml CII in the absence or presence of inhibitors of uptake for 30 minutes. Transmission electron microscopy was performed as described previously [28]. Briefly, cells were fixed in 2.5% EM grade gluteraldehyde (TAAB Lab. Equipment, Aldermaston, Berkshire, UK) diluted in 0.1 M phosphate buffer, pH 7.3, washed in phosphate buffer and post-fixed with 1% osmium tetroxide (Agar Scientific, Stansted, Essex, UK). Samples were sequentially dehydrated through a graded acetone series, impregnated with TAAB epoxy resin kit (TAAB Lab. Equipment) and polymerized at 60°C for 24 hours. Blocks were thin sectioned (80 nm), stained with uranyl acetate and lead citrate (Leica UK Ltd, Milton Keynes, UK), and examined with a Philips CM 100 (Compustage) Transmission Electron microscope (Philips Electron Optics, Eindhoven, The Netherlands). Sections through several planes of more than 50 APCs were examined for each treatment.

### Confocal microscopy

Bone-marrow macrophages and DCs were pulsed with 200 μg/ml CII in the absence or presence of 1.0 mM amiloride for 30 minutes at 37°C. Cytospins were prepared by centrifugation of 2 × 10^4 ^cells in 200 μl in a Shandon Cytospin 3 cytocentrifuge (Thermo Electron Corp., Waltham, MA, USA). The slides were air-dried at room temperature for 30 minutes, fixed in acetone for 10 minutes at room temperature and permeabilized in 0.1% Triton X-100 in PBS for 15 minutes at 4°C. After washing (10 mM TRIS HCl pH 7.6, containing 150 mM NaCl, TBS buffer) and blocking (normal rabbit serum 1:5 in TBS buffer, 1 hour at room temperature) staining was performed with goat anti-human CII polyclonal antibodies (1:100 in TBS buffer, 4°C, 18 hours; Santa Cruz Biotechnology, Inc.). Slides were washed and incubated with rabbit anti-goat IgG-FITC (1:100, 2 hours, room temperature, in the dark). After washing, slides were mounted in aqueous fluorescent mounting medium (DAKO Cytomation, Carpenteria, CA, USA). Confocal microscopy was performed at the BioImaging facility, University of Newcastle upon Tyne, using Leica TCS SP2 UV laser scanning confocal microscope (Leica Microsystems GmbH, Heidelberg, Germany) equipped with Time 63 oil immersion 1.32 No Plan A Pro lens. Images were acquired using the 488 excitation laser and emission was detected between 500 and 560 nm. Images were collected using 0.5 μm Z-steps and these were projected using maximal projection and overlaid with single optimized transmitted light images. In the control, cells were incubated in the absence of CII, stained and imaged at the same gain and offset levels as the positive cells and no fluorescence was observed.

## Results

### Mechanisms of CII uptake in macrophages and DCs

**Figure 1:**
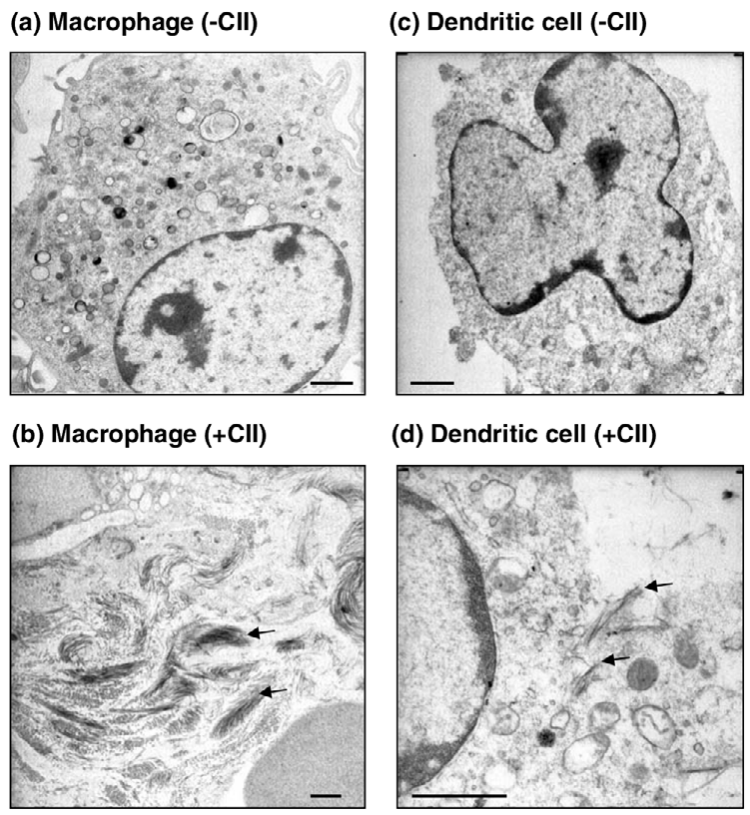
Electron micrographs of dendritic cells and macrophages pulsed with type II collagen (CII). **(a,b)** Macrophages and **(c,d)** dendritic cells were incubated in the (a,c) absence and (b,d) presence of 200 μg/ml CII for 30 minutes and analyzed by transmission electron microscopy. The arrows show fibrils of collagen aligned parallel to the plane of the section. Magnification: (a) ×8,900; (b) ×6,610; (c) ×8,900; (d) ×21,000. Bar = 1 μm. Sections through several planes of more than 50 cells were examined for each treatment.

To study the mechanisms of uptake of CII, macrophages and DCs from HLA-DR1-tg mice were incubated with CII for 30 minutes and visualized by transmission electron microscopy (Figure 1a–d). CII fibrils of different size were seen inside macrophages and DCs, showing that CII was internalized (Figure 1b,d). However, CII fibrils were rarely seen in the multiple sections examined, presumably because of the low probability of the plane section coinciding with the longitudinal axis of the CII fibrils.

**Figure 2:**
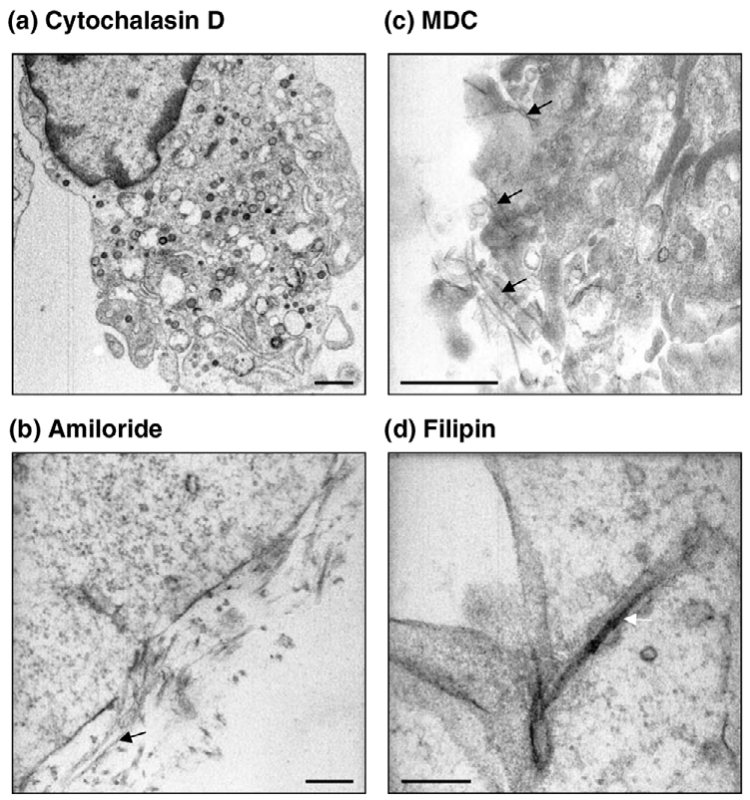
Electron micrographs of the effect of inhibitors of uptake on type II collagen (CII) internalization by macrophages. Macrophages were pulsed with 200 μg/ml CII for 30 minutes in the presence of **(a)** 10.0 μM cytochalasin D, **(b)** 1.0 mM amiloride, **(c)** 5.0 μM monodansylcadaverine (MDC) or **(d)** 0.4 μg/ml filipin and analyzed by electron microscopy. Magnification: (a) ×6,610; (b) ×52,000; (c) ×21,000; (d) ×73,000. Bar = (a,c) 1 μm or (b,d) 200 nm. Black arrows show fibrils of collagen aligned parallel to the plane of the section; the white arrow shows an unwinding collagen fibril inside the cell. Sections through several planes of more than 50 cells were examined for each treatment.

Electron microscopy studies also revealed that cytochalasin D, which prevents F-actin polymerization and hence inhibits both phagocytosis and macropinocytosis [34], blocked the appearance of CII inside both macrophages and DCs (Figure 2a). To distinguish between phagocytosis and macropinocytosis, cells were treated with amiloride, which inhibits membrane Na^+^/H^+^-ATPase, membrane ruffling and macropinocytosis [35]. Internalization of CII was also undetectable in the presence of amiloride (Figure 2b), suggesting the involvement of macropinocytosis rather than phagocytosis in the uptake of CII [28]. In contrast, monodansylcadaverine, which inhibits formation of clathrin-coated pits and subsequent receptor-mediated endocytosis [36], and filipin, which inhibits caveolae formation [37], did not prevent CII uptake (Figure 2c,d). These data suggest that CII was internalized by macrophages and DCs primarily by macropinocytosis.

**Figure 3:**
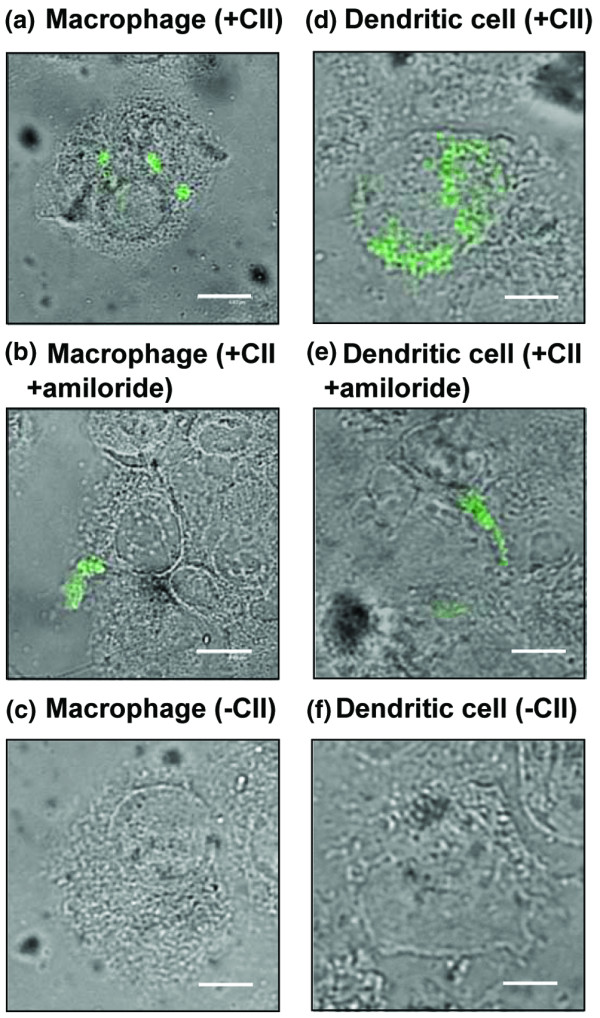
Confocal micrographs of dendritic cells and macrophages pulsed with type II collagen (CII). **(a-c)** Macrophages and **(d-f)** dendritic cells were incubated in the (a,b,d,e) presence or (c,f) absence of 200 μg/ml CII for 30 minutes, stained for CII expression and analyzed by confocal microscopy. Magnification ×630, and bars denote (a) 6.63 μm, (b) 8.09 μm, (c) 5.0 μm, (d) 4.27 μm, (e) 4.64 μm and (f) 4.0 μm. More than 50 cells were examined for each treatment.

We confirmed the identity of the material internalized by macrophages and DCs as CII by confocal microscopy using anti-CII antibodies (Figure 3a,d). Interestingly, DCs displayed a relatively stronger CII-specific fluorescence compared with macrophages, which is consistent with the higher efficiency of DCs as APCs compared with macrophages [38]. Amiloride completely blocked the intracellular appearance of CII in both macrophages and DCs, leading to the accumulation of CII at the cell surface (Figure 3b,e), which is in agreement with our electron microscopy data. No unspecific fluorescence was observed in control experiments in the absence of CII (Figure 3c,f).

### Subcellular localization of CII after uptake

**Figure 4:**
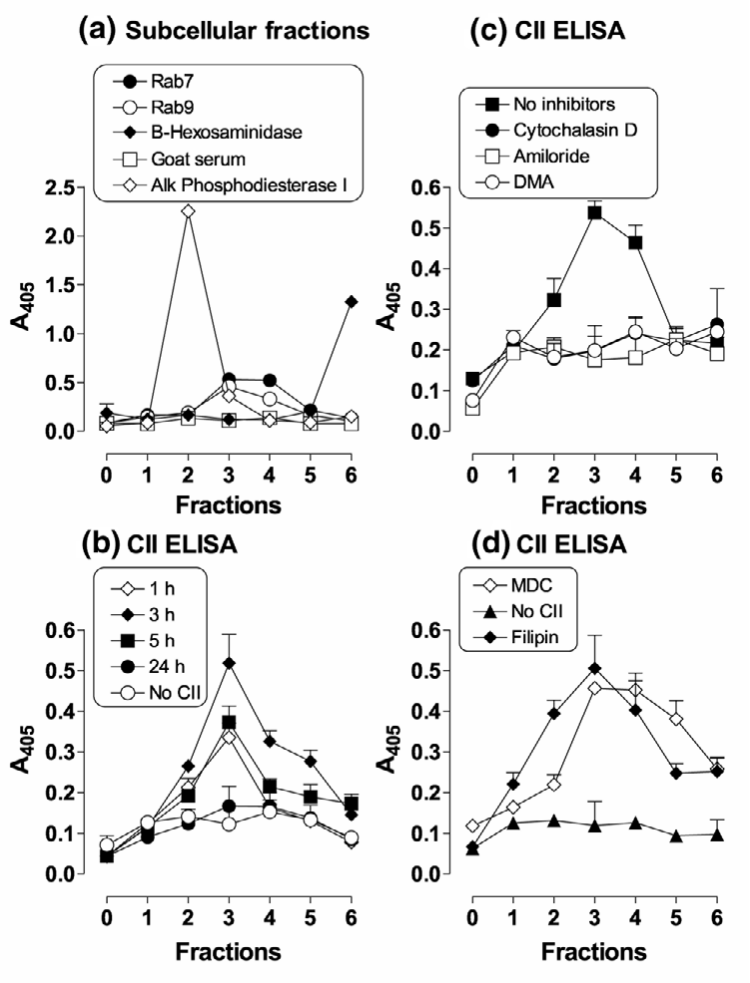
Subcellular distribution of type II collagen (CII) in macrophages. **(a)** Macrophages were subjected to subcellular fractionation and Percoll fractions were analyzed for the expression of the plasma membrane-associated enzyme alkaline phosphodiesterase I (open diamonds), the lysosomal enzyme β-hexosaminidase (closed diamonds) and markers of late endosomes Rab7 (closed circles) and Rab9 (open circles); 27% Percoll alone is shown as fraction 0. Enzyme activity was measured as absorbance at 405 nm. Goat serum was used as a negative control (squares). **(b)** Macrophages were incubated in the absence (open circles) or presence of 200 μg/ml CII for 30 minutes and chased for 1 (open diamonds), 3 (closed diamonds), 5 (squares) and 24 h (closed circles) followed by subcellular fractionation and CII-specific ELISA. **(c-d)** Macrophages were pulse-chased with CII as above: (c) in the absence (closed squares) or presence of cytochalasin D (closed circles), amiloride (open squares) and 5-(N,N-dimethyl)amiloride (DMA; open circles); (d) in the presence of monodansylcadaverine (MDC; open diamonds) and filipin (closed diamonds) in the doses shown in the legend to Figure 1 or in the absence of CII and inhibitors (triangles). Cells were subjected to subcellular fractionation followed by CII-specific ELISA. Absorbance was measured at 405 nm. One of two experiments showing essentially the same results is shown. Error bars denote standard deviation.

To establish the subcellular localization of CII after uptake, macrophages were subjected to subcellular fractionation by Percoll density gradient centrifugation, and subcellular fractions were analyzed for markers characteristic of different subcellular compartments. Alkaline phosphodiesterase I was localized only to fraction 2, indicating enrichment for plasma membranes [39], and the activity of the enzyme β-hexosaminidase was detected in dense membrane fraction 6 (Figure 4a), indicating localization of lysosomes [40]. As Rab7 and Rab9 GTPases have been shown to be associated with late endosomes and MHC class II loading compartments [41,42], we assayed Percoll fractions for Rab7 and Rab9 expression. Membrane fractions 3 and 4 with intermediate density (Figure 4a) expressed Rab7 and Rab9, indicating the presence of late endosomes including MHC class II loading compartments [43].

Macrophages were pulsed with 200 μg/ml CII for 30 minutes and chased for different periods of time. Following subcellular fractionation, the distribution of intracellular CII was measured by ELISA (Figure 4b). The intracellular level of CII peaked 3 hours after pulse and returned to the baseline after 24 hours. After internalization, CII was detected in Percoll fractions 3 and 4 with intermediate density co-localizing with Rab7 and Rab9 late endosomal markers. These pulse-chase experiments showed that after uptake CII was present for about five hours in membrane fractions corresponding to late endosomes, after which the level of intracellular CII dropped, probably due to terminal lysosomal transport and degradation.

We addressed the route of CII uptake into late endosomes in pulse-chase experiments in the presence of inhibitors of uptake. Pretreatment of macrophages with cytochalasin D or amiloride reduced accumulation of CII in fractions 3 and 4 (Figure 4c). Monodansylcadaverine and filipin had no effect on CII internalization (Figure 4d), consistent with data from electron microscopy (Figure 2c,d) and suggests internalization of CII primarily by macropinocytosis.

### Effect of uptake on activation of CII-specific T cells *in vitro*

We studied whether prevention of CII uptake by DCs and macrophages results in down-regulation of antigen presentation and inhibits activation of CII-specific T cells in *in vitro *antigen presentation assays. Since T cells specific for the glycosylated and non-glycosylated CII have been demonstrated in peripheral blood of RA patients [44,45], T cell hybridomas HCII-9.2, specific for the glycosylated C_259–273 _epitope, and HCII-9.1, specific for the non-glycosylated form of the same epitope, were used in this study [27].

**Figure 5:**
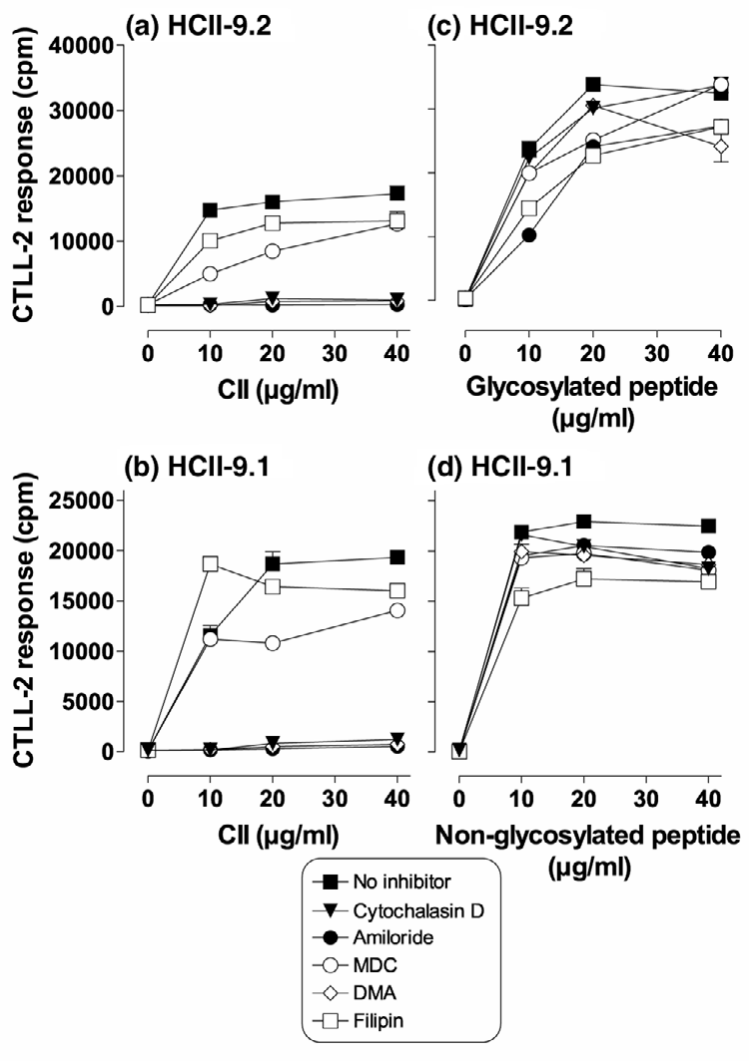
The effect of inhibitors of uptake on the intracellular processing of type II collagen (CII) by macrophages. Macrophages from HLA-DR1-tg mice were pulsed with a dilution series of **(a,b)** CII or **(c,d)** synthetic peptides in the absence (closed squares) or presence of cytochalasin D (triangles), amiloride (closed circles), 5-(N,N-dimethyl)amiloride (DMA; diamonds), monodansylcadaverine (MDC; open circles) or filipin (open squares) in the doses shown in the legend to Figure 1 for 5 hours. After fixation, plates were assayed with the (a,c) T cell hybridoma HCII-9.2 specific for the glycosylated epitope CII_259–273 _or (b,d) T cell hybridoma HCII-9.1 specific for the non-glycosylated form of the same epitope. IL-2 production by T cell hybridomas was assayed as proliferation of cytotoxic T cell line-2 (CTLL-2) cells in the presence of ^3^H-thymidine, and the results are presented as mean counts per minute (cpm) ± standard deviation (SD). A representative of three experiments is shown and error bars denote SD.

**Figure 6:**
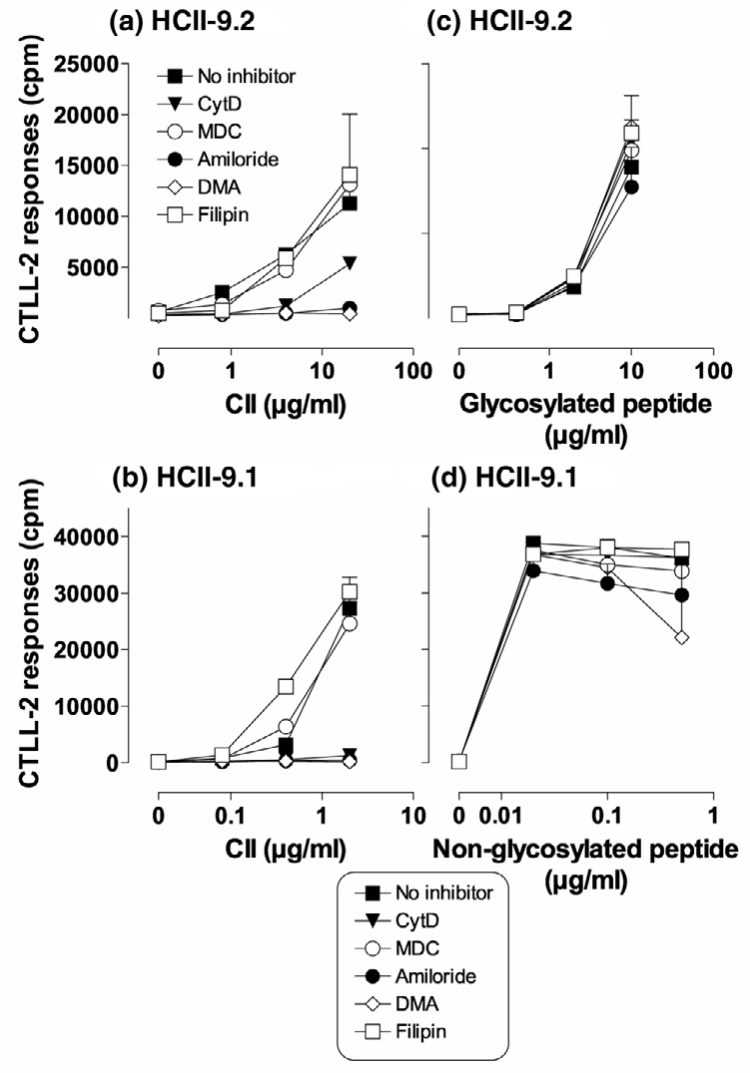
The effect of inhibitors of uptake on the intracellular processing of type II collagen (CII) by dendritic cells. Dendritic cells from HLA-DR1-tg mice were pulsed with a dilution series of **(a,b)** CII or **(c,d)** synthetic peptides in the absence (closed squares) or presence of cytochalasin D (triangles), amiloride (closed circles), 5-(N,N-dimethyl)amiloride (DMA; diamonds), monodansylcadaverine (MDC; open circles) or filipin (open squares) in the doses shown in the legend to Figure 1 for 5 hours. After fixation, plates were assayed with the (a,c) T cell hybridoma HCII-9.2 specific for the glycosylated epitope CII_259–273 _or (b,d) T cell hybridoma HCII-9.1 specific for the non-glycosylated form of the same epitope. Other details are as in the legend to Figure 4.

Macrophages were pulsed with CII or synthetic peptides in the absence or presence of inhibitors for 5 hours, fixed and assayed with T cell hybridomas HCII-9.2 and HCII-9.1. Macrophages were treated with cytochalasin D, which disrupts actin-mediated uptake, and amiloride to block membrane Na^+^/H^+^-ATPase, membrane ruffling and macropinocytosis. Both inhibitors markedly reduced presentation of CII to both T cell hybridomas (Figure 5a,b). Monodansylcadaverine and filipin, which interfere with clathrin-dependent and caveolin-dependent endocytosis, respectively, had no major effect on CII presentation (Figure 5a,b). We also confirmed the blocking effect of amiloride by using and the membrane-permeable derivative DMA (Figure 5a,b). Presentation of synthetic peptides was not significantly affected by the inhibitors used (Figure 5c,d). Antigen presentation by DCs was also inhibited by cytochalasin D, amiloride or DMA, but not by monodansylcadaverine or filipin (Figure 6a,b). Peptide presentation by DCs was not affected by inhibitors of uptake (Figure 6c,d). Amiloride and cytochalasin D used in this study as inhibitors of uptake have been also shown to inhibit activation of nuclear factor (NF)-κB and LPS-mediated DC maturation [46,47] Therefore, in separate antigen presentation experiments, immature DCs (not stimulated with LPS) were tested with inhibitors of uptake, and similar data were obtained (data not shown), suggesting that the effect of amiloride and cytochalasin D was independent of NF-κB inhibition. Dose-response data obtained in the absence of inhibitors presented in Figures 5 and 6 were also analyzed by the four parameter logistic equation to measure the dose of CII that causes 50% T cell hybridoma responses in antigen presentation assays (Effective Dose50, ED50). According to our calculations, DCs presented CII with about two-fold higher efficiency compared with macrophages and there was no difference between the glycosylated and non-glycosylated epitope presentation.

**Figure 7:**
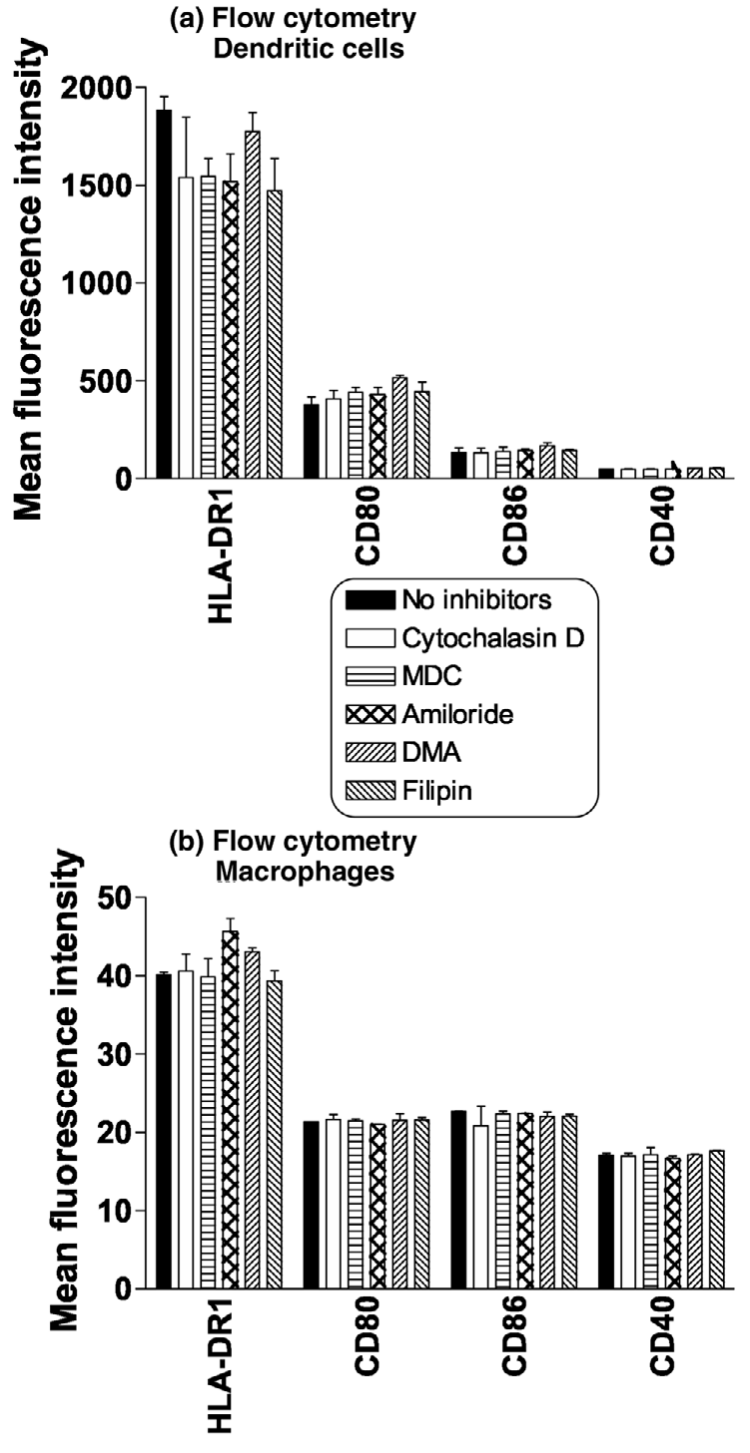
The effect of inhibitors of uptake on APC phenotype. **(a)** Dendritic cells or **(b)** macrophages were pretreated for 5 hours with cytochalasin D (open bars), monodansylcadaverine (MDC; ladder-hatched bars), 5-(N,N-dimethyl)amiloride (DMA; hatched bars), amiloride (cross-hatched bars) or filipin (back-hatched bars) in the doses shown in the legend to Figure 1 before preparation for flow cytometry. Data for the expression of HLA-DR1, CD40, CD80 and CD86 are shown as mean fluorescent intensity. No significant differences were detected for all inhibitors compared with untreated cells in three independent experiments by paired two-tailed *t* test (*P* > 0.05).

Mean fluorescence intensity analyzed by flow cytometry was used as an indicator of the level of expression of MHC class II and co-stimulatory molecules on the surface of macrophages and DCs. The expression of HLA-DR1, or CD40, CD80 and CD86 by macrophages and DCs was not significantly affected by inhibitors of uptake (Figure 7a,b). Similarly, the proportion of macrophages and DCs expressing these molecules on the cell surface was not significantly affected by the inhibitors used (data not shown). Therefore, the effect of inhibitors of uptake on antigen presentation and T cell activation was unlikely be due to expression of MHC class II or co-stimulatory molecules. The level of expression of HLA-DR1, CD80, CD86 and CD40 was higher in DCs compared with macrophages, which is consistent with the higher antigen presentation capacity of DCs.

### The effect of amiloride *in vivo*

**Figure 8:**
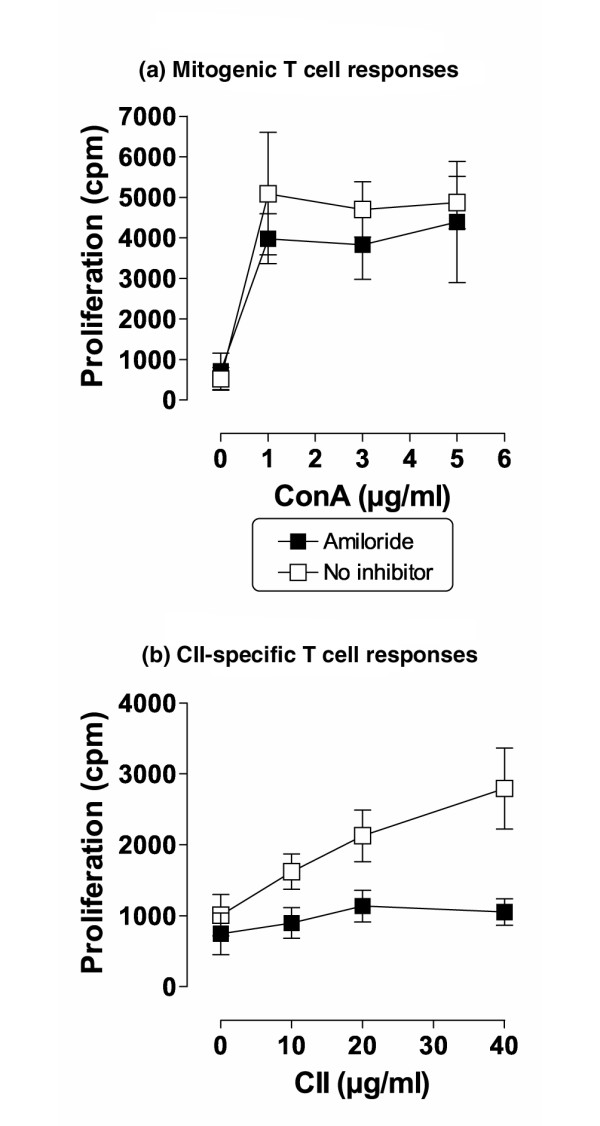
The effect of inhibitors of uptake on T cell proliferation *in vivo*. To test the effect of amiloride on mitogenic and type II collagen (CII)-specific T cell proliferation *in vivo*, groups of four mice were footpad immunized with CII emulsified in TiterMax adjuvant in the absence (no inhibitor) or presence of 150 μg/mouse amiloride (amiloride), and **(a)** mitogenic or **(b)** CII-specific T cell responses of the popliteal lymph node cells were assayed in triplicates 7 days later. Radioactivity incorporation was quantified as counts per minute (cpm) and cpm of cells alone was 797.6 (95% confidence interval from 643.7 to 951.4; *n *= 35). To show biological variation, mean data and error bars denoting 95% confidence interval are presented.

Our data show that pretreatment of professional APCs with amiloride prevents activation of CII-specific T cells *in vitro*. We also confirmed the effect of amiloride on CII-specific T cell responses *in vivo*. Mice were immunized with CII in adjuvant in the absence or presence of 150 μg/mouse of amiloride followed by assaying proliferation of popliteal lymph node cells 7 days later. The dose of amiloride was chosen based on the previously published doses used for *in vivo *treatment for other purposes [31]. T cell responses to concanavalin A were not affected by amiloride treatment (Figure 8a). A reduction in the CII-specific proliferative T cell responses in draining popliteal lymph nodes from mice immunized in the presence of amiloride was observed (Figure 8b), suggesting that CII uptake for presentation to T cells could be prevented *in vivo*.

## Discussion

We studied the mechanisms of uptake of CII by macrophages and DCs for presentation to T cells specific for the arthritogenic epitope CII_259–273_. Electron microscopy and antigen presentation to CII_259–273_-specific T and presentation cell hybridomas demonstrated that uptake of CII by both types of APCs depended on actin polymerisation (cytochalasin D-sensitive) and membrane ruffling (amiloride-sensitive), suggesting the principal route was macropinocytosis. Previous electron microscopy studies showed that fibroblasts use an F-actin-dependent mechanism for CII uptake, with no distinction between phagocytosis and macropinocytosis [48]. Macrophages have also been shown to have vacuoles containing collagen, suggesting their involvement in uptake and resorption of collagen [49]. However, no information was available on the capacity of other cell types to take up CII, as well as on the relevance of collagen uptake to antigen presentation and specific T cell activation. We extended the electron microscopy studies with pulse-chase experiments and localization of CII by subcellular fractionation and showed that after uptake, CII accumulated in membrane fractions with intermediate density corresponding to late endosomes. Moreover, blockade of macropinocytosis prevented intracellular accumulation of CII and resulted in profound blockade of antigen presentation to T cells. The involvement of macropinocytosis in uptake of autoantigens, such as CII, by both DCs and macrophages for subsequent antigen processing and presentation to specific T cells is a novel finding. Macropinocytosis has been previously shown to deliver antigens for lysosomal processing and loading of newly synthesized MHC class II molecules in DCs [50,51] and macrophages [28]. This observation is in agreement with our previous report that CII is processed in lysosomal compartments of macrophages for presentation by newly synthesized MHC class II molecules [27].

Our model system used CD4 T cell hybridomas specific for both the glycosylated and non-glycosylated arthritogenic epitope CII_259–273 _generated from HLA-DR1-transgenic mice [27], which allowed us to test the effect of post-translational modification on uptake and presentation of CII. No differential effect of the inhibition of uptake on presentation of the glycosylated and non-glycosylated CII_259–273 _epitope was observed. In a previous report we showed that glycosylated and non-glycosylated forms of the same CII_259–273 _epitope were differentially processed in lysosomal compartments for presentation to specific CD4 T cells [27]. Taken together, our data indicate that following macropinocytosis CII is targeted to lysosomes for antigen processing and presentation of both glycosylated and non-glycosylated epitopes to T cells. This conclusion is consistent with the presence of T cells specific for both forms of the epitope in peripheral blood of RA patients [44,45].

The importance of our finding that blockade of CII uptake prevents activation of specific T cells *in vitro *was tested *in vivo*. We administered amiloride *in vivo *and showed reduction in the magnitude of CII-specific, but not polyclonal, T cell responses in draining lymph nodes, suggesting that under these experimental conditions amiloride did not directly affect the T cell response, as has been reported in other experimental settings [52,53]. Our data suggest that amiloride caused an immunosuppressive effect on T cell activation *in vivo *indirectly via inhibition of uptake and antigen presentation, rather than via a direct suppression of T cell proliferation [52,53] Amiloride has also been shown to block soluble urokinase-type plasminogen activator [54], a serine proteinase expressed by macrophages and DCs (our unpublished observations), suggesting another mechanism underlying the effect of this drug on antigen presentation.

The potential of immunotherapeutic protocols based on the blockade of antigen presentation has been underscored in RA, including targeting co-stimulatory or MHC class II molecules [55,56] on APCs or T cell adhesion molecules on T cells [57], which has prompted the search for new ways of down-regulating antigen presentation *in vivo*. The results of this study suggest that interfering with antigen uptake could constitute a novel effective target for blocking antigen presentation in DCs and macrophages, as a way to prevent activation of specific CII-specific T cells. The data obtained have implications for the development of immunotherapeutic protocols for use in T cell-mediated autoimmune diseases, such as RA.

## Conclusion

This study shows that macropinocytosis was the predominant mechanism of uptake of CII for antigen presentation by DCs and macrophages. Treatment of both professional APC types with amiloride, which prevents macropinocytosis, inhibited intracellular accumulation of CII and antigen presentation of the major arthritogenic T cell epitope in both glycosylated and non-glycosylated forms. In addition, treatment of mice with amiloride blocked the activation of collagen-specific T cells in draining lymph nodes, constituting a novel therapeutic target for the immunotherapy of RA.

## Abbreviations

APC = antigen-presenting cell; CII = type II collagen; DC = dendritic cell; DMA = 5-(N,N-dimethyl)amiloride; ELISA = enzyme-linked immunosorbent assay; FBS = fetal bovine serum; LPS = lipopolysaccharide; mAb = monoclonal antibody; MHC = major histocompatibility complex; NF = nuclear factor; RA = rheumatoid arthritis.

## Competing interests

The authors declare that they have no competing interests.

## Authors' contributions

AvD was involved in study design, and was responsible for data acquisition, analysis and interpretation as well as manuscript preparation. CMUH, CVH, DMA, NM, HR, JDI and RH contributed to study design and data analysis and interpretation. JHR was responsible for study design, data analysis and interpretation, as well as manuscript preparation. All authors read and approved the final manuscript.
